# Association between genetic variants of membrane transporters and the risk of high-grade hematologic adverse events in a cohort of Mexican children with B-cell acute lymphoblastic leukemia

**DOI:** 10.3389/fonc.2023.1276352

**Published:** 2024-01-10

**Authors:** Deyanira Escalante-Bautista, Doris Cerecedo, Elva Jiménez-Hernández, Carolina González-Torres, Javier Gaytán-Cervantes, Juan Carlos Núñez-Enríquez, Omar Alejandro Sepúlveda-Robles, Marlon De Ita, Silvia Jiménez-Morales, José Manuel Sánchez-López, Minerva Mata-Rocha, José Refugio Torres-Nava, Jorge Alfonso Martín-Trejo, Luz Victoria Flores-Villegas, María de Lourdes Gutiérrez-Rivera, Laura Elizabeth Merino-Pasaye, Karina Anastacia Solís-Labastida, María Raquel Miranda-Madrazo, Gabriela Alicia Hernández-Echáurregui, Darío Orozco-Ruíz, Janet Flores-Lujano, María Luisa Pérez-Saldívar, Juan Manuel Mejía-Aranguré, Haydeé Rosas-Vargas

**Affiliations:** ^1^ Unidad de Investigación Médica en Genética Humana, Hospital de Pediatría “Dr. Silvestre Frenk Freund”, Centro Médico Nacional Siglo XXI, Instituto Mexicano del Seguro Social, Ciudad de México, Mexico; ^2^ Laboratorio de Hematobiología, Escuela Nacional de Medicina y Homeopatía, Instituto Politécnico Nacional, Ciudad de México, Mexico; ^3^ Servicio de Oncohematología Pediátrica, Hospital Pediátrico Moctezuma, Secretaría de Salud de la Ciudad de México, Ciudad de México, Mexico; ^4^ Universidad Autónoma Metropolitana, Unidad Xochimilco, Ciudad de México, Mexico; ^5^ Escuela Superior de Medicina, Instituto Politécnico Nacional, Ciudad de México, Mexico; ^6^ Laboratorio de Secuenciación, División de Desarrollo de la Investigación, Centro Médico Nacional Siglo XXI, Instituto Mexicano del Seguro Social, Ciudad de México, Mexico; ^7^ Unidad de Investigación Médica en Epidemiología Clínica, Hospital de Pediatría, Centro Médico Nacional Siglo XXI, Instituto Mexicano del Seguro Social, Ciudad de México, Mexico; ^8^ Laboratorio de Innovación y Medicina de Precisión, Núcleo A, Instituto Nacional de Medicina Genómica, Ciudad de México, Mexico; ^9^ Servicio de Oncología, Hospital Pediátrico de Moctezuma, Secretaría de Salud de la Ciudad de México, Ciudad de México, Mexico; ^10^ Servicio de Hematología Pediátrica, Hospital de Pediatría “Dr. Silvestre Frenk Freund”, Centro Médico Nacional Siglo XXI, Instituto Mexicano del Seguro Social, Ciudad de México, Mexico; ^11^ Servicio de Hematología Pediátrica, Centro Médico Nacional “20 de Noviembre”, Instituto de Seguridad y Servicios Sociales de los Trabajadores del Estado, Ciudad de México, Mexico; ^12^ Servicio de Oncología Pediátrica, Hospital de Pediatría “Dr. Silvestre Frenk Freund”, Centro Médico Nacional Siglo XXI, Instituto Mexicano del Seguro Social, Ciudad de México, Mexico; ^13^ Laboratorio de Genómica del Cáncer, Instituto Nacional de Medicina Genómica, Ciudad de México, Mexico; ^14^ Facultad de Medicina, Universidad Nacional Autónoma de México, Ciudad de México, Mexico

**Keywords:** pharmacogenomics, membrane transporters, toxicity, acute lymphoblastic leukemia, ATP-binding cassette transporter, solute carrier family, hematologic adverse event

## Abstract

**Background:**

Advances in the understanding of the pathobiology of childhood B-cell acute lymphoblastic leukemia (B-ALL) have led towards risk-oriented treatment regimens and markedly improved survival rates. However, treatment-related toxicities remain a major cause of mortality in developing countries. One of the most common adverse effects of chemotherapy in B-ALL is the hematologic toxicity, which may be related to genetic variants in membrane transporters that are critical for drug absorption, distribution, and elimination. In this study we detected genetic variants present in a selected group genes of the *ABC* and *SLC* families that are associated with the risk of high-grade hematologic adverse events due to chemotherapy treatment in a group of Mexican children with B-ALL.

**Methods:**

Next generation sequencing (NGS) was used to screen six genes of the ABC and seven genes of the SLC transporter families, in a cohort of 96 children with B-ALL. The grade of hematologic toxicity was classified according to the National Cancer Institute’s Common Terminology Criteria for Adverse Events (CTCAE) version 5.0, Subsequently, two groups of patients were formed: the null/low-grade (grades 1 and 2) and the high-grade (grades 3 to 5) adverse events groups. To determine whether there is an association between the genetic variants and high-grade hematologic adverse events, logistic regression analyses were performed using co-dominant, dominant, recessive, overdominant and log-additive inheritance models. Odds ratio (OR) and 95% confidence intervals (95% CI) were calculated.

**Results:**

We found two types of associations among the genetic variants identified as possible predictor factors of hematologic toxicity. One group of variants associated with high-grade toxicity risk: *ABCC1* rs129081; *ABCC4* rs227409; *ABCC5* rs939338, rs1132776, rs3749442, rs4148575, rs4148579 and rs4148580; and another group of protective variants that includes *ABCC1* rs212087 and rs212090; *SLC22A6* rs4149170, rs4149171 and rs955434.

**Conclusion:**

There are genetic variants in the *SLC* and *ABC* transporter families present in Mexican children with B-ALL that can be considered as potential risk markers for hematologic toxicity secondary to chemotherapeutic treatment, as well as other protective variants that may be useful in addition to conventional risk stratification for therapeutic decision making in these highly vulnerable patients.

## 1 Introduction

Acute Lymphoblastic Leukemia (ALL) is the predominant form of cancer in children (1). According to its cellular lineage, ALL is classified as T-cell ALL (T-ALL) and B-cell ALL (B-ALL); the latter type is the most frequent and comprises 80%-85% of ALL patients (2). Despite the existence of efficient treatment protocols for childhood ALL, 75% of patients experience therapy-related adverse effects, and 5% die from treatment toxicity (3–5). Among the most frequent side effects are hematologic adverse events leading to immunosuppression and myelosuppression and, consequently, an increased risk of infections (6, 7). Moreover, the effects of chemotherapy toxicity lead to treatment modifications or discontinuations which compromise the outcome (8–10).

The processes of drug transport and cellular detoxification involve the so-called ABC transporters, which comprise a superfamily of proteins that use ATP to move substrates across membranes (11). This superfamily consists of 49 members that are divided into 7 subfamilies from A to G according to sequence similarity, domain organization, and by function, whether importing or exporting (12, 13). The transport mechanisms in which these proteins participate are essential for the uptake of drugs through the intestinal wall, their expulsion from the tissues into the systemic circulation and, finally, their elimination through the kidneys and liver. Particularly, within this family of transporters, ABCC1-6 are involved in the transport of chemotherapeutic agents, such as adriamycin, vincristine, etoposide, 6-mercaptopurine, and methotrexate (14–16).

In addition, there are the SLC transporters that represent a superfamily with more than 400 members. Like the ABC family, they are classified into subfamilies according to sequence similarity (17). Their functions include a role in drug absorption and excretion, especially the SLC22 and SLCO subfamilies that are predominantly expressed in kidney, liver, and intestine. There is a broad consensus on the importance of these transporters in the mechanisms concerning the pharmacokinetics and pharmacodynamics of drugs used in chemotherapy, as well as the significance of genetic variants that alter their structure and function. As particular examples, the SLC22A6, SLC22A8 genes have been identified as involved in the methotrexate pathway and some variants in these genes are associated with a delay in their clearance (18, 19).

Some pharmacogenetic tests are now available to help in detecting key variants related to response and side effects to chemotherapy treatment in B-ALL patients. This information represents a valuable tool in making therapeutic decisions aimed at reducing the risk of toxicity before starting chemotherapy. However, the routinely analyzed variants are limited, and a high prevalence of toxicity is still observed in patients with B-ALL (20, 21). Furthermore, it is important to note that the frequency of some of the variants associated with an increased risk of toxicity in Caucasian population may differ in populations of a different genetic background such as the Hispanics. Taking the above into account, the aim of this study was to detect genetic variants present in a selected group genes of the *ABC* and *SLC* families that are associated with the risk of high-grade hematologic adverse events due to chemotherapy treatment in a group of Mexican children with B-ALL.

## 2 Materials and methods

### 2.1 Patients and samples

The study included ninety-six pediatric patients with B-ALL, diagnosed between 2014 and 2019 in public hospitals, members of the Mexican Interinstitutional Group for the Identification of the Causes of Childhood Leukemia (MIGICCL). These hospitals provide treatment to about 95% of pediatric patients with leukemia in Mexico City. The diagnosis of B-ALL was made at each institution by bone marrow aspirate for histopathological analysis and immunophenotyping. Peripheral blood samples were obtained at the end of remission induction. The pre-analytical sample processing route was performed by trained personnel assigned to each step following official Mexican regulations. The personnel assigned to each hospital attended every day to collect blood samples and/or bone marrow aspirate from the patients; and these were immediately transported to the laboratory where they were processed on the same day of collection, separating the different fractions. For this work, genomic DNA was extracted from the leukocyte fraction and, once the nucleic acids were extracted, the samples were stored properly labeled with a format to preserve patient anonymity in a biobank at -70°C until their use. The sample identification data and clinical characteristics of the patient are integrated in both physical and digital records. Patients were treated using one of two protocols, modified HP09 ALL Berlin-Frankfurt-Münster 95 protocol or St. Jude Total Therapy XIIIB modified (**Supplementary Tables S1** , **S2**), depending on the hospital in which they were assisted. All biological samples and clinical information were obtained under written informed consent signed by parents and informed assent from children aged ≥ 8 years. A structured and exhaustive review of the medical records was performed to capture information related to hematologic toxicity from admission onwards. In case of death, the death certificate was consulted. The correlation between genetic variants and risk of hematologic toxicity was performed in the period of the first 100 days, from the beginning of therapy to the end of the consolidation phase of treatment, since during this period the differences in the physiological response to chemotherapy are clearer because these are the most intense phases of treatment. The grade of severity of each adverse event (AE) was defined according to the Common Terminology Criteria for Adverse Events (CTCAE), version 5, of the National Cancer Institute (22). Five severity event categories were considered: grade 1 (mild), clinical or diagnostic observations, asymptomatic laboratory signs or mild symptoms requiring no intervention; grade 2 (moderate), requiring minimal, local or noninvasive interventions; grade 3 (severe), requiring or prolonging hospitalization or incapacitating; grade 4 (life-threatening), when urgent medical intervention was required; and grade 5 (death), when death was related to the AE. Based on this classification two comparative groups were formed; the first,named as the null/low-grade adverse event group, included patients with no manifestations of hematologic toxicity during the established period as well as patients with adverse events grade 1 or 2. The second, termed high-grade adverse events group, consisted of patients with grade 3 and 4 with episodes of severe and life-threatening hematologic toxicity, respectively; and the only deceased patient in category 5 whose death was due to the hemato-toxic effects of chemotherapy. The total number of events per group is shown in **Supplementary Figure S1**. This study was approved by the ethics and scientific boards of the Mexican Institute of Social Security (IMSS) (registration number R-2015-785-121).

### 2.2 DNA extraction

DNA was extracted from peripheral blood samples using Maxwell^®^ 16 Blood DNA Purification Kit (Promega, Madison, WI, USA) according to manufacturer’s recommendations. The purity and concentration of the DNA samples were measured using a Qubit 4 fluorometer (Thermo Fisher Scientific, Waltham, MA, USA).

### 2.3 Data collection

Information regarding sex, age at diagnosis, white blood cells (WBC) count in peripheral blood at diagnosis confirmation, immunophenotype, gene rearrangement characterization, and chemotherapeutic scheme was collected from patient records by personnel with prior standardized training. The four most common gene rearrangements with a known impact on the prognosis of children with leukemia (*ETV6*::*RUNX1*, *TCF3*::*PBX1*, *BCR*::*ABL1*, and *KMT2A*::*AFF1*), were examined by RT-PCR. Risk classification was determined at the time of diagnosis according to the NCI criteria: standard risk (ages from 1-9.99 years; WBC count <50,000/μl) or as high risk (age <1 or ≥10 years or WBC ≥50,000/μl).

### 2.4 Next-generation sequencing

Variants of the genes of interest were detected by next-generation sequencing (NGS) with a custom panel (Agilent, Santa Clara, CA, USA) to target coding regions, UTRs and some intronic variants of the transporters of the ABC and SLC family that had been previously reported as risk variants for hematologic toxicity: *ABCC1, ABCB1, ABCC2, ABCC4, ABCC5, ABCG2, SLC19A1, SLC29A1, SLC22A1, SLC28A3, SLC22A8, SLC22A6* and *SLC25A37*. Capture-based enriched libraries were pooled (12 indexed libraries per pool) and sequenced on a Nextseq 500 System (Illumina, San Diego, CA, USA). Sequencing depth coverage was 60 to 100 X for all target regions. Genetic variant detection was analyzed with the Genome Analysis Toolkit (GATK, Broad Institute, Cambridge, UK).

### 2.5 Statistical analysis

The clinical characteristics of the two groups, with null/low or high toxicity, were compared either by chi square test (two-sided) or Fisher’s Exact Test when expected values were less than five, using SPSS Statistics 24.0 (IBM Canada Ltd.). *P*-values ≤0.05 were considered statistically significant. Deviation from Hardy–Weinberg equilibrium (HWE) of genetic variants was calculated based on chi-square test. Association analysis between single nucleotide variants (SNVs) and hematologic adverse events grade under five models of inheritance (codominant, dominant, recessive, overdominant, and log-additive model) were performed using online SNPStats program (http://bioinfo.iconcologia.net/SNPStats). The strength of association was measured by odds ratio (OR) with a 95% confidence interval (CI). The best model of inheritance for each SNV was selected based on Akaike (AIC) and Bayessian (BIC) information criteria. Linkage disequilibrium (LD) and haplotypes were analyzed using the online program SNPStats, and the LD plot was generated in Haploview version 4.1 software.

## 3 Results

### 3.1 Patients general characteristics

A total of 96 patients with B-ALL aged less than 16 years of age were included in the present study (**Table 1**). Of these, 44 were female and 52 were male. In terms of child´s sex, age, leukocyte count at baseline, chemotherapy protocol and risk classification, no statistically significant differences were found between groups. Fusion transcripts were not detected in most patients, and in 11 cases these data were not available. Most of the adverse events of chemotherapy were detected in the consolidation phase.

**Table 1:** Clinical features of the study population according to hematologic adverse events grade classification in patients B- ALL.

	Null/Low-Grade AE N (%)	High-Grade AE N (%)	*p-value*
Total	34 (35.4)	62 (64.2)	
Child´s sex, n (%)			
Female	15 (15.63)	29 (30.21)	0.48
Male	19 (19.79)	33 (34.37)	
Age at diagnosis			
1-9.9 years	22 (22.92)	35 (36.46)	0.28
≥10 years	12 (12.5)	27 (28.12)	
WBC count in peripheral blood			
<50,000/µL	28 (29.17)	47 (48.96)	0.31
≥50,000/µL	6 (6.25)	15 (15.62)	
NCI risk			
Standard	21 (21.88)	27 (28.13)	0.06
High	13 (13.54)	35 (36.45)	
Chemotherapy protocol			
SJT XIIIB	25 (26.04)	40 (41.67)	0.25
BFM-95	9 (9.38)	22 (22.91)	
Grade of adverse event			
Null	17 (17.70)	0	<0.001
Grade 1	7 (7.29)	0	
Grade 2	10 (10.42)	0	
Grade 3	0	18 (18.75)	
Grade 4	0	43 (44.79)	
Grade 5	0	1 (1.05)	
Type of gene rearrangement, n (%)			
Not assessed/not detected	5/25 (31.26)	6/49 (57.29)	0.56
*E2A::PBX1*	2 (2.08)	2 (2.08)	
*ETV6::RUNX1*	2 (2.08)	2 (2.08)	
*BCR::ABL1*	0	3 (3.13)	

AE, Adverse Events; NCI, National Cancer Institute risk classification; WBC count in peripheral blood; St. Jude Total XIIIB, BFM- 95, Berlin-Frankfurt-Munster 95. *p* value calculated by Chi square tests/Fisher’s exact test. A value of *p* ≤ 0.05 was considered statistically significant.

### 3.2 Genetic variants associated with risk of high-grade hematologic adverse events

**Figure 1:**
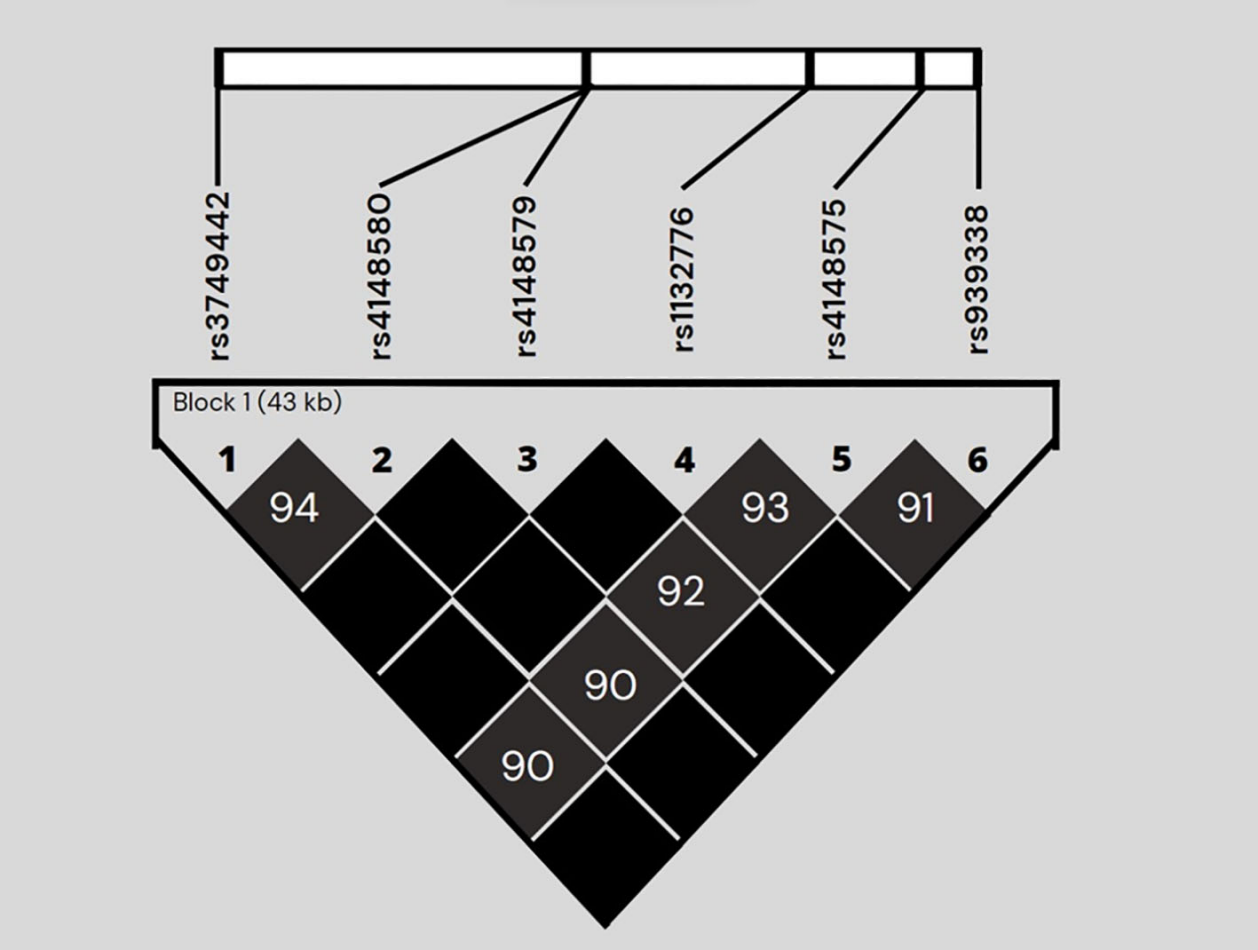
Linkage disequilibrium plot generated by Haploview software of the genetic risk variants of the *ABCC5* gene. Linkage disequilibrium (LD) is shown as pairwise D' values. The unnumbered black diamonds correspond to D' values of 1.0.

Through NGS screening, some variants were detected in the *ABC* and *SLC* gene families that were associated with the risk of developing hematologic toxicity events of high grade. This association was analyzed using codominant, dominant, recessive, overdominant and log-additive models of inheritance (**Supplementary Table S3**). Selection of the inheritance model was based on Akaike and Bayesian informative criteria (data not shown). All genotype frequencies were in accordance with the Hardy-Weinberg equation. **Table 2** shows the frequencies of variants detected with a statistically significant association with an increased risk of high-grade adverse events. The adjustment of our correlation analysis was performed considering the effect of homocedasticity and the effect of gender, age at diagnosis, white blood cell count at baseline, and genetic rearrangements. All detected risk variants belong to the *ABCC* gene family (**Supplementary Table S4**); two of them under the dominant model: *ABCC1* rs129081, OR=2.58, 95% CI=1.01-6.57, p=0.047; and *ABCC4* rs2274409, OR=2.9, 95% CI=1.16-7.27, p=0.014 (**Table 2**). Additionally, a risk association was found with a group of *ABCC5* variants (r3749442; rs4148580; rs4148579; rs4148575 and rs939338), all of them under the recessive model. Among these *ABCC5* variants it is noteworthy that genotypes rs3749442 AA (p=0.0066) and rs4148580 TT (p=0.019) are present only in individuals in the high-grade adverse events group. We then analyzed the linkage disequilibrium (LD) of the *ABCC5* variants group using Haploview 4.1. The pairwise D’ values obtained are higher than 0.9 (p<0.001), so we can confirm that these nine variants of the *ABCC5* gene are commonly inherited as a block (**Figure 1**). On the other hand, in this same gene we found four variants with OR values of 7.12 p=0.021, but broad confidence intervals (CI) ranging from 0.88 to 57.70 (**Supplementary Table S5**); therefore, although a trend is observed as risk variants, statistical significance was not obtained. For the *SLC* transporter family, no statistically significant association was found between any of its variants and the risk of high-grade hematologic adverse events.

**Table 2:** Association of high-grade hematologic adverse events risk with genetic variants in *ABC* and *SLC* genes.

Genetic Variant	Inheritance Model	Genotype	Null/Low-Grade AE N (%)	High-Grade AE N (%)	OR (95% CI)	*p-*value
*ABCC1* rs129081	Dominant	G/G	13 (38.2%)	12 (19.4%)	1.00	0.047
		G/C-C/C	21 (61.8%)	50 (80.7%)	2.58 (1.01-6.57)	
*ABCC4* rs2274409	Dominant	C/C	23 (67.7%)	28 (45.2%)	1.00	0.014
		C/T-T/T	11 (32.4%)	34 (54.8%)	2.90 (1.16-7.27)	
*ABCC5* rs939338	Recessive	G/G -A/G	28 (82.3%)	38 (61.3%)	1.00	0.028
		A/A	6 (17.6%)	24 (38.7%)	2.95 (1.06-8.17)	
*ABCC5* rs1132776	Recessive	A/A-A/G	30 (88.2%)	43 (69.3%)	1.00	0.031
		G/G	4 (11.8%)	19 (30.6%)	3.31 (1.02-10.73)	
*ABCC5* rs3749442	Recessive	G/G -A/G	34 (100%)	54 (87.1%)	1.00	0.000
		A/A	0 (0%)	8 (12.9%)	NA (0.00-NA)	
*ABCC5* rs4148575	Recessive	A/A-A/G	28 (82.3%)	38 (61.3%)	1.00	0.028
		G/G	6 (17.6%)	24 (38.7%)	2.95 (1.06-8.17)	
*ABCC5* rs4148579	Recessive	T/T-T/C	30 (88.2%)	43 (69.3%)	1.00	0.031
		C/C	4 (11.8%)	19 (30.6%)	3.31 (1.02-10.73)	
*ABCC5* rs4148580	Recessive	T/T-T/C	34 (100%)	56 (90.3%)	1.00	0.019
		C/C	0 (0%)	6 (9.7%)	NA (0.00-NA)	

AE, Adverse Events; OR adjusted by sex, age at diagnosis, WBC count in peripheral blood, gene rearrangement, and chemotherapy protocol. A value of *p* ≤0.05 was considered statistically significant. NA, not applicable.

### 3.3 Genetic variants protective of high-grade hematologic adverse events

Along with the risk variants, we found protective variants in genes of both gene families under a dominant model of inheritance (**Table 3**, **Supplementary Table S4**). The rs212087 and rs212090 variants of the *ABCC1* gene, showed a protective association (OR=0.32, 95% CI=0.14-0.93, p=0.03; and OR=0.36, 95% CI=0.15-0.99, p=0.044, respectively). For the *SLC* family, a protective effect was observed from the rs4149170 and rs4149171 variants of the *SLC22A6* gene in linkage disequilibrium (pairwise D’ value 1), and a value of OR=0.32, 95% CI=0.12-0.91, p=0.030 in both cases. In the same gene, the rs955434 variant was detected as protective for high-grade hematologic adverse events (OR=0.37, 95% CI=0.14, p=0.040) in the study population.

**Table 3:** Protective variants for high-grade hematologic adverse events in *ABC* and *SLC* genes.

Genetic Variant	Inheritance Model	Genotype	Null/Low-grade AE N (%)	High-Grade AE N (%)	OR (95% CI)	*p-*value
*ABCC1* rs212087	Dominant	G/G	8 (23.5%)	31 (50%)	1	0.01
		G/A-A/A	26 (76.5%)	31 (50%)	0.32 (0.12-0.83)	
*ABCC1* rs212090	Dominant	T/T	9 (26.5%)	32 (51.6%)	1	0.016
		T/A- A/A	25 (73.5%)	30 (48.4%)	0.34 (0.14-0.84)	
*SLC22A6* rs4149170	Dominant	C/C	20 (58.8%)	49 (79%)	1	0.038
		C/T-T/T	14 (41.2%)	13 (21%)	0.38 (0.15-0.95)	
*SLC22A6* rs4149171	Dominant	TT	20 (58.8%)	49 (79%)	1	0.038
		T/C-CC	14 (41.2%)	13 (21%)	0.38 (0.15-0.95)	
*SLC22A6* rs955434	Dominant	G/G	17 (50%)	47 (75.8%)	1	0.011
		G/A-A/A	17 (50%)	15 (24.2%)	0.32 (0.13-0.78)	

AE, Adverse Events; OR adjusted by sex, age at diagnosis, WBC count in peripheral blood, gene rearrangement and chemotherapy protocol. A value of *p* ≤0.05 was considered statistically significant.

## 4 Discussion

Childhood type B acute lymphoblastic leukemia is the main type of childhood cancer in our country and, although survival rates have increased significantly in developed countries, in Mexico there is still a high mortality rate, part of which is associated with the adverse effects of chemotherapeutic treatment (23, 24). The individual’s ability to metabolize chemotherapeutic drugs depends on numerous factors including environment, gender, age, nutritional status, and genetic profile (25). The latter is of great relevance as it can cause changes in the structure of enzymes involved in drug metabolism and transport. These interindividual differences are the cause of treatment-related toxicities in a fraction of the pediatric ALL population, which can affect different organs and can range from mild to life-threatening (26). Currently, the application of FDA-approved pharmacogenetic testing has yielded promising results and encourages physicians to prescribe individualized medications (27). Nevertheless, a high percentage of patients continue to experience the effects of toxicity associated with chemotherapy treatment, the most frequent being hematologic toxicity (26, 27). Currently, the most successful example of pharmacogenetics in ALL in a clinical setting is the detection of genetic variants of genes encoding TPMT and NUDT enzymes prior to the prescription of thiopurine drugs and, although it has been implemented in different parts of our country, hematologic toxicity still occurs with high frequency (28). Since most of the studies related to this topic have been performed in Caucasian populations with different genetic backgrounds, it is essential to carry out pharmacogenomic studies in other populations. Therefore, in the present work, the main interest was to describe the association of genetic variants present in genes encoding drug transporters with high-grade hematologic adverse events caused by chemotherapeutic treatment in Mexican children diagnosed with B-ALL.

The uptake of chemotherapeutic treatment drugs used by blast cells may be affected by different transporters, including upstream and downstream transporters, especially solute transporters (SLC) and ATP-binding cassette (ABC) family pumps, respectively. Previous pharmacogenetic studies have suggested that single nucleotide variants of SLC and ABC transporters may play a promising role in drug exposure and have been associated with clinical response and toxicity. ABC transporters harness energy from ATP hydrolysis and function as efflux transporters, whereas SLC transporters are mainly involved in small molecule uptake into cells (29). In this study, we analyzed, by NGS, thirteen genes from these families selected based on the background of their association with risk of drug toxicity in the treatment of B-ALL. From this analysis we detected this type of association between *ABC* family variants and hematologic toxicity as risk factors in our group of B-ALL patients, particularly in *ABCC1*, *ABCC4* and *ABCC5*.

ABCC1 transports a wide range of antineoplastic agents, including methotrexate, anthracyclines, vinca alkaloids, and numerous glucuronidated, glutathionylated, and sulfated derivative agents. *ABCC1* gene sequence is highly conserved, however, genetic variants in its sequence have been associated with neurotoxicity, cardiotoxicity, and hematologic toxicity (30, 31). Vulsteke et al. (2013), reported the association of three variants in the *ABCC1* gene with hematologic toxicity during chemotherapy in a Belgian population of women with breast cancer (32). In this study, we found that the *ABCC1* rs129081 variant is associated with risk of high-grade hematologic toxicity. On the other hand, we observed that the *ABCC1* rs212087 and rs212090 variants are protective against high-grade adverse events. When contrasting these results with other populations, we found data confirming the presence of risk or protective variants in *ABCC1*. In this sense, Gutiérrez-Camino et al. (2018) reported that the rs3743527 variant was associated with protection against anthracycline cardiotoxicity in Mexican pediatric patients with ALL treated with a traditional chemotherapeutic regimen (33). On the other hand, Kunadt et al. (2020) analyzed the presence of *ABCC1* rs129081, rs212090 and 212091 variants located in the 3′ UTR region, finding an association with overall survival and disease-free survival in patients with AML. Although they did not conduct functional analyses, they propose that the change of nucleotides in these non-coding regions modifies the binding capacity of regulatory miRNAs with consequences on mRNA and protein expression (34). Thus, we can observe the duality between the presence of different variants in the same gene associated alternatively with risk or protection against high-grade hematological adverse events, which seem to influence the different responses to chemotherapeutic treatment.

Regarding the pharmacogenomics of the *ABCC4* gene, the association of certain variants with 6-mercaptopurine intolerance has been described in Chinese children with ALL, while an association with hematologic toxicity was found in an Egyptian population with ALL (35, 36). Similarly, we found a risk association between high-grade hematologic adverse events and the *ABCC4* rs2274409 variant. Besides, Krishnamurthy et al. (2008) observed that the rs3765534 missense variant reduces the function of the transporter by preventing its membrane localization, causing an accumulation of 6-thioguanine nucleotides in myeloid precursor cells (37). Although a possible negative dominant effect of this variant was ruled out, it was shown in a subsequent study in a Japanese population that the combination of rs3765534 with other *NUDT15* intermediate activity variants in ALL patients caused intolerance to 6-mercaptopurine and marked leukopenia (38). Therefore, in future studies it will be important to intentionally search for combinations of risk variants that may be particularly affecting our population.

As for ABCC5, it is an efflux transporter for cyclic nucleotides and nucleotide analogues (39). Some of its genetic variants have previously been associated with doxorubicin cardiotoxicity in survivors of childhood ALL in a cohort of Canadian patients (40). In this study, we observed a clear association between high-grade hematologic adverse events and six *ABCC5* gene variants, all in linkage disequilibrium. In addition, we found four more variants with a trend as risk factors, however, the confidence intervals are outside the limits of what can be considered a statistically significant association. In particular, the rs939336 variant stands out, which generates a stop codon that translates into a truncated protein that could affect its function in the transport of antineoplastic drugs (**Supplementary Table S4**). We consider that with a larger sample size it is very likely that this association between *ABCC5* variants and hematologic toxicity will be confirmed; in any case, the results obtained already reflect the importance of this gene in the pharmacogenomics of B-ALL in Mexican pediatric population.

Regarding the *SLC* family, it has been demonstrated in other populations that certain variants are associated with the risk of different manifestations of toxicity (41). Although several genes of the family were considered within the sequencing strategy of this work, we only observed a protective association against high-grade hematologic adverse events in the *SLC22A6* rs4149170, rs4149171 and rs955434 variants. To our knowledge, this is the first report of such an association with these specific variants. These results are in addition to those described by Visscher et al. (42), who demonstrated an association between the genetic variant rs7853758 of the *SLC28A3* gene as a protective factor against anthracycline-induced cardiotoxicity in the pediatric population. Although the most clinically relevant approach in the study of genetic variants usually focuses on the description of risk variants, it is also important to have information on protective variants.

The major limitation of this study was the small sample size. In a next phase, we plan to increase the number of patients and, additionally, to analyze the ancestry of the participants from different regions of the country. Equally important, it will be to complement this information with the detection of genetic variants clearly associated with hematological toxicity such as those of the *TPMT* and *NUDT15* genes. The objective is to have enough information to integrate it into a risk stratification algorithm according to the genetic background of each population and finally to be able to offer a personalized treatment with the lowest possible risk of side effects.

## 5 Conclusions

In summary, we found genetic variants within genes of the *SLC* and *ABC* transporter families as risk markers for high-grade hematologic adverse events risk from chemotherapeutic treatment of ALL in Mexican children, as well as other protective variants. These results show us that interindividual patient variability in these drug transporter coding genes should be considered in the management of their treatment as part of a personalized medicine strategy to improve the likelihood of a successful outcome in ALL patients.

## Data availability statement

The datasets presented in this study can be found in online repositories. The names of the repository/repositories and accession number(s) can be found below: https://www.ncbi.nlm.nih.gov/, PRJNA1004576.

## Ethics statement

The studies involving humans were approved by Comité Nacional de Investigación Científica del Instituto Mexicano del Seguro Social. The studies were conducted in accordance with the local legislation and institutional requirements. Written informed consent for participation in this study was provided by the participants’ legal guardians/next of kin.

## Author contributions

DE-B: Writing – original draft, Data curation, Formal analysis, Investigation, Methodology. DC: Formal analysis, Writing – original draft, Conceptualization, Supervision, Writing – review & editing. EJ-H: Conceptualization, Writing – review & editing, Funding acquisition. CG-T: Conceptualization, Writing – review & editing, Data curation, Formal analysis, Investigation, Methodology. JG-C: Data curation, Formal analysis, Investigation, Methodology, Writing – review & editing, Conceptualization. JN-E: Conceptualization, Data curation, Formal analysis, Investigation, Methodology, Writing – review & editing, Funding acquisition, Project administration, Supervision, Writing – original draft. OS-R: Formal analysis, Supervision, Writing – review & editing. MI: Formal analysis, Supervision, Writing – review & editing. SJ-M: Formal analysis, Supervision, Writing – review & editing. JS-L: Formal analysis, Supervision, Writing – review & editing. MM-R: Writing – review & editing, Conceptualization, Investigation, Methodology. JT-N: Investigation, Writing – review & editing. JM-T: Investigation, Writing – review & editing. LF-V: Investigation, Writing – review & editing. MG-R: Investigation, Writing – review & editing. LM-P: Investigation, Writing – review & editing. KS-L: Investigation, Writing – review & editing. MM-M: Investigation, Writing – review & editing. GH-E: Investigation, Writing – review & editing. DO-R: Investigation, Writing – review & editing. JF-L: Investigation, Writing – review & editing, Data curation. MP-S: Investigation, Writing – review & editing. JM-A: Writing – review & editing, Conceptualization, Formal analysis, Funding acquisition, Methodology, Project administration, Supervision, Writing – original draft. HR-V: Conceptualization, Formal analysis, Funding acquisition, Methodology, Project administration, Supervision, Writing – original draft, Writing – review & editing.

## Supplementary material

Supplementary Figure 1 Total number of hematologic adverse events recorded by group. **(A)** High-grade adverse events group, **(B)** Low-grade or no adverse events group. The overall number of events by grade and type according to CTCAE v5.0 is shown.
