# Supplementary figures and images for: Association between genetic variants of membrane transporters and the risk of high-grade hematologic adverse events in a cohort of Mexican children with B-cell acute lymphoblastic leukemia
Source: Front Oncol. 2024 Jan 10;13:1276352. doi: 10.3389/fonc.2023.1276352 (PMC10807790; doi:10.3389/fonc.2023.1276352)

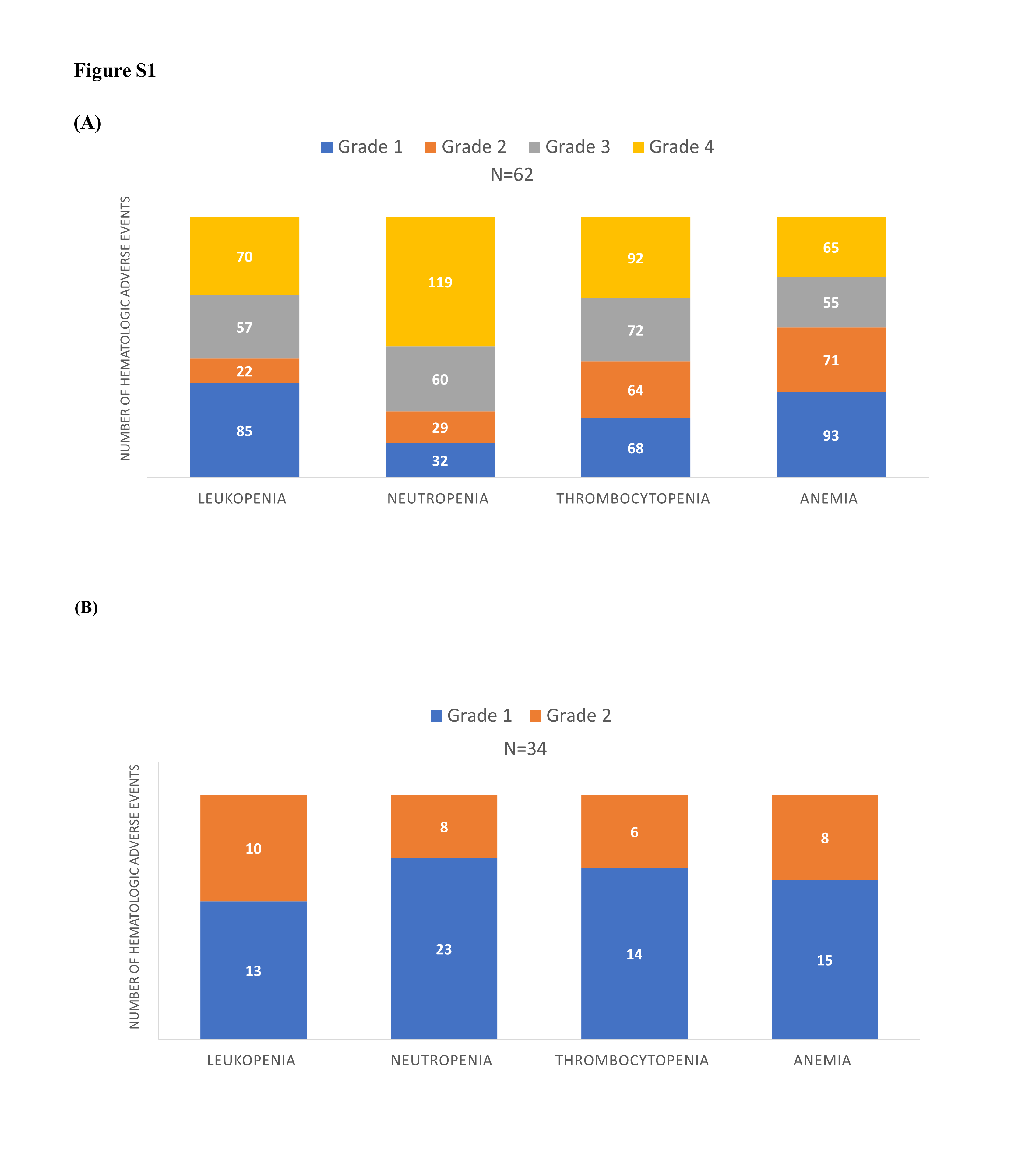

Supplement: Supplementary Figure 1 — Total number of hematologic adverse events recorded by group. (A) High-grade adverse events group, (B) Low-grade or no adverse events group. The overall number of events by grade and type according to CTCAE v5.0 is shown. [file Image_1.tif]
