# Supplementary material for: Association between genetic variants of membrane transporters and the risk of high-grade hematologic adverse events in a cohort of Mexican children with B-cell acute lymphoblastic leukemia
Source: Front Oncol. 2024 Jan 10;13:1276352. doi: 10.3389/fonc.2023.1276352 (PMC10807790; doi:10.3389/fonc.2023.1276352)
Supplement: Supplementary file 2 [file Table_1.pdf]

Table S1 Modified HP09 ALL Berlin-Frankfurt-Münster 95 protocol, from induction to consolidation.

Standard risk		
**Treatment**	**Single or daily dose**	**Day of application**
**Induction/Consolidation, Protocol I**		
**Phase A**		
Prednisone (PO)	60 mg/m² per day	1-28
Vincristine (IV)	1.5 mg/m² per day	1, 8, 15, 22
Daunorubicin (PI over 1 hour)	30 mg/m² per day	8, 15
L-asparginase (PI over 1 hour)	5000 IU/m² per dose	12. 15, 18, 21, 24, 27, 30, 33
Triple therapy (IT)	Dosage by age range*	1, 12, 33
**Consolidation**		
**Phase B**		
6-mercaptopurine (PO)	60 mg/m² per day	36-63
Cyclophosphamide (IV)	1000 mg/m² per dose	36, 64
Cytarabine (IV)	75 mg/m² per dose	38-41, 45-48, 52-55, 59-62
Triple therapy (IT)	Dosage by age range*	45, 59
**Phase C (Extracompartment therapy)**		
Methotrexate	1500 mg/m² per day	8, 22, 36, 50
6-mercaptopurine	25 mg/m² per day	1-56
Triple therapy (IT)	Dosage by age range*	8, 22, 36, 50
**Intensive Consolidation**		
**Phase D**		
Prednisone (PO)	60 mg/m² per day	1-28
Vincristine (IV)	1.5 mg/m² per day	1, 8, 15, 22
Daunorubicin (PI over 1 hour)	30 mg/m² per day	8, 15
L-asparaginase (PI over 6 hours)	5000 IU/m² per day	1, 8, 15, 22
Triple therapy (IT)	Dosage by age range*	1, 15,22
**High risk**		
**Treatment**	**Single or daily dose**	**Day of application**
**Induction/consolidation, protocol I**		
**Phase A**		
Prednisone (PO)	60 mg/m² per day	1-28
Vincristine (IV)	1.5 mg/m² per day	1, 8, 15, 22
Daunorubicin (PI over 1 hour)	30 mg/m² per day	8, 15,22, 29
L-asparaginase (PI over 1 hour)	5000 IU/m² per dose	12, 15, 18, 21, 24, 27, 30, 33
Triple therapy (IT)	Dosage by age range*	1, 12. 33
**Consolidation (Phase 1)**		
Dexamethasone (IV)	20 mg/m² per day	1-5
Methotrexate (PI)	1500 mg/m² per day, continuous infusion for 24 hours, divided into 2 doses	1
Cyclophosphamide (PI over 1 hour)	200 mg/m² per dose (5 doses, 12-hours intervals)	2-4
Cytarabine (PI)	1500 mg/m² per day, continuous infusion for 3 hours, every 12 hours	5
L-asparaginase (IM)	25000 IU/m² per day	6
Triple therapy (IT)	Dosage by age range*	1
Monocyte-granulocyte colony-stimulating factor	300 mcg	11, 12, 13, 14,15
**Consolidation (Phase 2)**		
Dexamethasone (IV)	30 mg/m² per day	1-5
Vincristine (IV)	1.5 mg/m² per day	1, 6
Daunorubicin (IV)	30 mg/m² per day	5

Ifosfamide (IV)	500 mg/m² per dose (5 doses, 12 hours intervals)	2-4
L-asparaginase (IM)	25000 IU/m² per dose	6
Triple therapy (IT)	Dosage by age range*	1
Monocyte-granulocyte colony-stimulating factor	300 mcg	10-14
**Consolidation (Phase 3)**		
Dexamethasone (PO)	30 mg/m² per day	1-5
Cytarabine (PI over 3 hours)	1500 mg/m² per day (4 doses, 12 hours intervals)	1-2
Etoposide (PI over 1 hour)	100 mg/m² per day	3-5 (5 doses, 12-hour intervals)
L-asparaginase (IM)	25000 UI/m² dose	6
Triple therapy (IT)	Dosage by age range*	5
Monocyte-granulocyte colony-stimulating factor	300 mcg	11-15
**The three consolidation phases are repeated.**		
**Intensification**		
Prednisone (PO)	60 mg/m² per day	28 days
Vincristine (IV)	1.5 mg/m² per dose (max 2 mg)	1, 8, 15, 22
Daunorubicin (IV)	30 mg/m² day	8, 15, 21, 29
L-asparaginase (IM)	10 000 IU/m² dose	8, 11, 15, 18

PO indicates orally; IV, intravenous push; PI, intravenous infusion; IT, intrathecally; IM, intramuscular; IU, international units.

*Intrathecal triple chemotherapy medications calculated according to the patient´s age:

Methotrexate (MTX): < 1 year: 6 mg; 1-3 years: 8 mg; 3-4 years: 10 mg; >4 years: 12 mg; >12 years: 15 mg

Hydrocortisone: < 1 year: 12 mg; 1-3 years: 16 mg; ≥3 years: 25 mg

Cytarabine: < 1 year: 15 mg; 1-3 years: 20 mg; 3-9 years: 25 mg; >9 years: 30 mg

¶ From end of intensive chemotherapy until 120 weeks after diagnosis.

Table S2 St. Jude Total Therapy XIIIB modified, from induction to consolidation.

Standard risk		
**Treatment**	**Single dose or daily dose**	**Day of application**
**Induction/consolidation, protocol I**		
**Phase A**		
Prednisone (PO)	60 mg/m² per day	0-28
Vincristine (IV)	2 mg/m² per day	7, 14, 21, 28
Daunorubicin (PI)	30 mg/m² per day (one-hour infusion)	7, 14
L-asparaginase IM	10,000 UI/m² per dose	6, 8, 10, 12, 14, 16
Triple therapy (IT)	Dosage by age range*	0, 7, 14, 21
**Intensification**		
**Phase B**		
Etoposide (PI)	300 mg/m² per dose (two-hours infusion)	28, 33, 38
Cytarabine (PI)	300 mg/m² per dose (four-hours infusion)	28, 33, 38
**Consolidation**		
**Phase C**		
6-Mercaptopurine (PO)	60 mg/m² per day	26-39
Cyclophosphamide (PI)	1000 mg/m² per dose (One- hour infusion)	36, 64
Cytarabine (PI)	75 mg/m² per dose (One-hour infusion)	27-30, 34-37
Triple therapy (IT)	Dosage by age range*	29, 43
Pre-hydration	Glucose solution 5%, 400 ml/m² + 40 mEQ of NaHCO3/L	Two-hour infusion prior to the start of methotrexate
Hydration	Glucose solution 5%, 3000 ml/m² / 24 hrs + 40 mEQ de NaHCO3/L	
Methotrexate (PO)	2 g/m² per day	29, 43
**High risk**		
**Treatment**	**Single dose or daily dose**	**Day of application**
**Induction/consolidation, protocol I**		
**Phase A**		
Prednisone (PO)	60 mg/m² per day	0-7
Vincristine (IV)	2 mg/m² per day	7, 14, 21, 28
Daunorubicin (PI)	30 mg/m² per day (one-hour infusion)	7, 14, 21, 28
L-asparaginase IM	10,000 IU/m² per dose	6, 8, 10, 12, 14, 16
Triple therapy (IT)	Dosage by age range*	0, 7, 14, 21
Cyclophosphamide (PI)	1000 mg/m² per day (one-hour infusion)	26
**Intensification**		
**Phase B**		
Etoposide (PI)	300 mg/m² per day (two-hours infusion)	28, 33, 38
Cytarabine (PI)	300 mg/m² per day (four-hours infusion)	28, 33, 38
**Consolidation**		
**Phase C**		
6-Mercaptopurine (PO)	60 mg/m² per day	26-39
Cyclophosphamide (PI)	1000 mg/m² per dose (one-hour infusion)	36, 64
Cytarabine (PI)	75 mg/m² per dose (one-hour infusion)	27-30 and 34-37
Pre-hydration	Glucose solution 5%, 400 ml/m² + 40 mEQ of NaHCO3/L	Two-hour infusion prior to the start of methotrexate
Hydration	Glucose solution 5% a 3000 ml/m²/ 24 hrs + 40 mEQ de NaHCO3/L	

Methotrexate (PO)	5 g/m² per day	1, 15, 29, 43

PO indicates orally; IV, intravenous push; PI, intravenous infusion; IT, intrathecally; IM, intramuscular; IU, international units.

*Intrathecal triple chemotherapy medications calculated according to the patient´s age:
Methotrexate (MTX): < 1 year: 6 mg; 1-2 years: 8 mg; 2-3 years: 10 mg; >3 years: 12 mg
Hydrocortisone: < 1 year: 12 mg; 1-2 years: 16 mg; 2-3 years: 20 mg; >3 years: 24 mg
or Dexamethasone: < 1 year: 4 mg; 1-2 years: 4 mg; 2-3 years: 5 mg; >3 years: 5 mg
Cytarabine: < 1 year: 18 mg; 1-2 years: 24 mg; 2-3 years: 30 mg; >3 years: 36 mg

Table S3 Association analysis of variants in the *ABC* and *SLC* genes with high-grade hematologic adverse events under five inheritance models.

	Inheritance Model	Genotype	Null/Low-Grade AE N (%) 34 (35.4)	High-Grade AE N (%) 62 (64.6)	OR (95% CI)	*P* value
***ABCC1* rs129081**	Codominant	G/G	13 (38.2%)	12 (19.4%)	1	0.049
		G/C	18 (52.9%)	35 (56.5%)	2.11 (1.04-5.55)	
		C/C	3 (8.8%)	15 (24.2%)	5.42 (1.25-23.49)	
	Dominant	G/G	13 (38.2%)	12 (19.4%)	1	0.047
		G/C-C/C	21 (61.8%)	50 (80.7%)	2.58 (1.01-6.57)	
	Recessive	G/G-G/C	31 (91.2%)	47 (75.8%)	1	0.053
		C/C	3 (8.8%)	15 (24.2%)	3.30 (1.08-12.34)	
	Overdominant	G/G-C/C	16 (47.1%)	27 (43.5%)	1	0.740
		G/C	18 (52.9%)	35 (56.5%)	1.15 (0.50-2.67)	
	Log-additive	---	---	---	2.17 (1.05-4.49)	0.064
***ABCC1* rs212087**	Codominant	G/G	8 (23.5%)	31 (50%)	1	0.015
		G/A	18 (52.9%)	26 (41.9%)	0.38 (0.14-1.04)	
		A/A	8 (23.5%)	5 (8.1%)	0.18 (0.04-0.71)	
	Dominant	G/G	8 (23.5%)	31 (50%)	1	0.010
		G/A-A/A	26 (76.5%)	31 (50%)	0.32 (0.12-0.83)	
	Recessive	G/G-G/A	26 (76.5%)	57 (91.9%)	1	0.039
		A/A	8 (23.5%)	5 (8.1%)	0.29 (0.09-0.96)	
	Overdominant	G/G-A/A	16 (47.1%)	36 (58.1%)	1	0.300
		G/A	18 (52.9%)	26 (41.9%)	0.64 (0.28-1.49)	
	Log-additive	---	---	---	0.40 (0.21-0.76)	0.390
***ABCC1* rs212090**	Codominant	A/A	9 (26.5%)	32 (51.6%)	1	0.026
		T/A	18 (52.9%)	25 (40.3%)	0.39 (0.15-1.02)	
		T/T	7 (20.6%)	5 (8.1%)	0.20 (0.05-0.79)	
	Dominant	A/A	9 (26.5%)	32 (51.6%)	1	0.016
		T/A-T/T	25 (73.5%)	30 (48.4%)	0.34 (0.14-0.84)	
	Recessive	A/A -T/A	27 (79.4%)	57 (91.9%)	1	0.083
		T/T	7 (20.6%)	5 (8.1%)	0.34 (0.10-1.16)	
	Overdominant	A/A-T/T	16 (47.1%)	37 (59.7%)	1	0.230
		T/A	18 (52.9%)	25 (40.3%)	0.60 (0.26-1.40)	
	Log-additive	---	---	---	0.43 (0.23 – 0.83)	0.920
***ABCC4* rs2274409**	Codominant	C/C	23 (67.7%)	28 (45.2%)	1	0.048
		C/T	10 (29.4%)	27 (43.5%)	2.55 (0.97-6.71)	
		T/T	1 (2.9%)	7 (11.3%)	5.89 (0.65-53.16)	
	Dominant	C/C	23 (67.7%)	28 (45.2%)	1	0.014
		C/T-T/T	11 (32.4%)	34 (54.8%)	2.90 (1.16-7.27)	
	Recessive	C/T-C/T	33 (97.1%)	55 (88.7%)	1	0.130
		T/T	1 (2.9%)	7 (11.3%)	4.30 (0.49-37.83)	
	Overdominant	C/C-T/T	24 (70.6%)	35 (56.5%)	1	0.170
		C/T	10 (29.4%)	27 (43.5%)	1.85 (0.76-4.52)	
	Log-additive	---	---	---	2.29 (1.09-4.80)	0.210
***ABCC5* rs939338**	Codominant	G/G	8 (23.5%)	12 (19.4%)	1	0.088
		G/A	20 (58.8%)	26 (41.9%)	0.87 (0.30-2.52)	
		A/A	6 (17.6%)	24 (38.7%)	2.67 (0.75-9.45)	
	Dominant	G/G	8 (23.5%)	12 (19.4%)	1	0.630
		G/A-A/A	26 (76.5%)	50 (80.7%)	1.28 (0.47-3.53)	
	Recessive	G/G-G/A	28 (82.3%)	38 (61.3%)	1	0.028
		A/A	6 (17.6%)	24 (38.7%)	2.95 (1.06-8.17)	
	Overdominant	G/G-A/A	14 (41.2%)	36 (58.1%)	1	

		G/A	20 (58.8%)	26 (41.9%)	0.51 (0.22-1.18)	0.110
	Log-additive	---	---	---	1.65 (0.91-3.01)	0.097
***ABCC5* rs1132776**	Codominant	A/A	9 (26.5%)	14 (22.6%)	1	0.094
		A/G	21 (61.8%)	29 (46.8%)	0.89 (0.32-2.43)	
		G/G	4 (11.8%)	19 (30.6%)	3.05 (0.78-11.96)	
	Dominant	A/A	9 (26.5%)	14 (22.6%)	1	0.670
		A/G-G/G	25 (73.5%)	48 (77.4%)	1.23 (0.47-3.25)	
	Recessive	A/A-A/G	30 (88.2%)	43 (69.3%)	1	0.031
		G/G	4 (11.8%)	19 (30.6%)	3.31 (1.02-10.73)	
	Overdominant	A/A-G/G	13 (38.2%)	33 (53.2%)	1	0.160
		A/G	21 (61.8%)	29 (46.8%)	0.54 (0.23-1.28)	
	Log-additive	---	---	---	1.62 (0.87-3.02)	0.120
***ABCC5* rs3749442**	Codominant	G/G	18 (52.9%)	27 (43.5%)	1	0.024
		G/A	16 (47.1%)	27 (43.5%)	1.12 (0.48-2.66)	
		A/A	0 (0%)	8 (12.9%)	NA (0.00-NA)	
	Dominant	G/A	18 (52.9%)	27 (43.5%)	1	0.380
		G/A-A/A	16 (47.1%)	35 (56.5%)	1.46 (0.63-3.38)	
	Recessive	G/G-G/A	34 (100%)	54 (87.1%)	1	0.006
		A/A	0 (0%)	8 (12.9%)	NA (0.00-NA)	
	Overdominant	G/G-A/A	18 (52.9%)	35 (56.5%)	1	0.740
		G/A	16 (47.1%)	27 (43.5%)	0.87 (0.37-2.01)	
	Log-additive	---	---	---	1.79 (0.89-3.61)	0.095
***ABCC5* rs4148575**		A/A	8 (23.5%)	13 (21%)	1	0.081
	Codominant	A/G	20 (58.8%)	25 (40.3%)	0.77 (0.27-2.22)	
		G/G	6 (17.6%)	24 (38.7%)	2.46 (0.70-8.64)	
	Dominant	A/A	8 (23.5%)	13 (21%)	1	0.770
		A/A-A/G	26 (76.5%)	49 (79%)	1.16 (0.43-3.16)	
	Recessive	A/A-A/G	28 (82.3%)	38 (61.3%)	1	0.028
		G/G	6 (17.6%)	24 (38.7%)	2.95 (1.06-8.17)	
	Overdominant	A/A-G/G	14 (41.2%)	37 (59.7%)	1	0.082
		A/G	20 (58.8%)	25 (40.3%)	0.47 (0.20-1.11)	
	Log-additive	---	---	---	1.58 (0.88-2.85)	0.120
***ABCC5* rs4148579**	Codominant	T/T	9 (26.5%)	13 (21%)	1	0.097
		T/C	21 (61.8%)	30 (48.4%)	0.99 (0.36-2.73)	
		C/C	4 (11.8%)	19 (30.6%)	3.29 (0.83-12.98)	
	Dominant	T/T	9 (26.5%)	13 (21%)	1	0.540
		T/C-C/C	25 (73.5%)	49 (79%)	1.36 (0.51-3.60)	
	Recessive	T/T-T/C	30 (88.2%)	43 (69.3%)	1	0.031
		C/C	4 (11.8%)	19 (30.6%)	3.31 (1.02-10.73)	
	Overdominant	T/T-C/C	13 (38.2%)	32 (51.6%)	1	0.210
		T/C	21 (61.8%)	30 (48.4%)	0.58 (0.25-1.36)	
	Log-additive	---	---	---	1.70 (0.90-3.20)	0.093
***ABCC5* rs4148580**	Codominant	T/T	20 (58.8%)	29 (46.8%)	1	0.053
		T/C	14 (41.2%)	27 (43.5%)	1.33 (0.56-3.15)	
		C/C	0 (0%)	6 (9.7%)	NA (0.00-NA)	
	Dominant	T/T	20 (58.8%)	29 (46.8%)	1	0.260
		T/C-C/C	14 (41.2%)	33 (53.2%)	1.63 (0.70-3.79)	
	Recessive	T/T-T/C	34 (100%)	56 (90.3%)	1	0.019
		C/C	0 (0%)	6 (9.7%)	NA (0.00-NA)	
	Overdominant	T/T – C/C	20 (58.8%)	35 (56.5%)	1	0.820
		T/C	14 (41.2%)	27 (43.5%)	1.10 (0.47-2.57)	
	Log-additive	---	---	---	1.86 (0.89-3.88)	0.090
	Codominant	C/C	20 (58.8%)	49 (79%)	1	0.110
		C/T	13 (38.2%)	12 (19.4%)	0.38 (0.15-0.97)	
		T/T	1 (2.9%)	1 (1.6%)	0.41 (0.02-6.85)	

***SLC22A6* rs4149170**	Dominant	C/C	20 (58.8%)	49 (79%)	1	0.038
		C/T – T/T	14 (41.2%)	13 (21%)	0.38 (0.15-0.95)	
	Recessive	C/C-C/T	33 (97.1%)	61 (98.4%)	1	0.670
		T/T	1 (2.9%)	1 (1.6%)	0.41 (0.02-6.85)	
	Overdominant	C/C-T/T	21 (61.8%)	50 (80.7%)	1	0.470
		C/T	13 (38.2%)	12 (19.4%)	0.54 (0.03-8.93)	
	Log-additive	---	---	---	0.44 (0.19-1.00)	0.058
***SLC22A6* rs4149171**	Codominant	T/T	20 (58.8%)	49 (79%)	1	0.110
		T/C	13 (38.2%)	12 (19.4%)	0.38 (0.15-0.97)	
		C/C	1 (2.9%)	1 (1.6%)	0.41 (0.02-6.85)	
	Dominant	T/T	20 (58.8%)	49 (79%)	1	0.038
		T/C-C/C	14 (41.2%)	13 (21%)	0.38 (0.15-0.95)	
	Recessive	T/T-T/C	33 (97.1%)	61 (98.4%)	1	0.067
		T/T	1 (2.9%)	1 (1.6%)	0.54 (0.03-8.93)	
	Overdominant	T/T-C/C	21 (61.8%)	50 (80.7%)	1	0.470
		T/C	13 (38.2%)	12 (19.4%)	0.39 (0.15-0.99)	
	Log-additive	---	---	---	0.44 (0.19-1.00)	0.058
***SLC22A6* rs955434**	Codominant	G/G	17 (50%)	47 (75.8%)	1	0.030
		G/A	16 (47.1%)	13 (21%)	0.29 (0.12-0.74)	
		A/A	1 (2.9%)	2 (3.2%)	0.72 (0.06-8.50)	
	Dominant	G/G	17 (50%)	47 (75.8%)	1	0.011
		G/A-A/A	17 (50%)	15 (24.2%)	0.32 (0.13-0.78)	
	Recessive	G/G-G/A	33 (97.1%)	60 (96.8%)	1	0.95
		A/A	1 (2.9%)	2 (3.2%)	1.10 (0.10-12.59)	
	Overdominant	G/G-A/A	18 (52.9%)	49 (79%)	1	0.85
		G/A	16 (47.1%)	13 (21%)	0.30 (0.12-1.04)	
	Log-additive	---	---	---	0.43 (0.20-0.93)	0.0

AE, Adverse Events; OR adjusted by sex, age at diagnosis, WBC count in peripheral blood, gene rearrangement and chemotherapy protocol. A value of p ≤ 0.05 was considered statistically significant.

Table S4 Summary of *ABCC* and *SLC* variants linked to risk or protection to high-grade adverse hematologic events in B-ALL patients.

Genetic variant	Variant position and change	Functional consequence	MAF
**Risk variants**			
ABCC1 **rs129081**	NG_028268.2:g.197506G>A	3´ UTR variant	C=0.442
ABCC4 **rs2274409**	NC_000013.10:g.95860214C>T	Intron	T=0.2028
ABCC5 **rs939338**	NC_000003.11:g.183704068G>A	Intron	A=0.5464
ABCC5 **rs1132776**	NG_047115.1:g.44397A>G	Synonymous	G=0.5318
ABCC5 **rs3749442**	NC_000003.11:g.183660585G>A	Synonymous	A=0.2362
ABCC5 **rs4148575**	NC_000003.11:g.183702275A>G	Non coding transcript variant	G=0.5497
ABCC5 **rs4148579**	NC_000003.11:g.183685249T>C	Intron	C=0.5323
ABCC5 **rs4148580**	NC_000003.11:g.183685183T>C	Intron	C=0.1814
**Protective variants**			
ABCC1 **rs212087**	NG_028268.2:g.191857G>A	Intron	A=0.4036
ABCC1 **rs212090**	NG_028268.2:g.197571T>A	3’ UTR Variant	A=0.412
SLC22A6 **rs4149170**	NC_000011.9:g.62752289C>T	5’ UTR Variant	T=0.1251
SLC22A6 **rs4149171**	NC_000011.9:g.62752182T>A	5’UTR Variant	A=0.000
SLC22A6 **rs955434**	NC_000011.9:g.62757113G>A	Undefined	A=0.2079

MAF; Minor allele frequency in Latin American individuals with mostly European and Native American Ancestry according to ALFA Allele Frequency, release version: 20230706150541

Table S5. Genetic variants in the *ABCC5* gene showing a trend as risk factors for high-grade hematologic adverse events.

	Inhereitance Model	Genotype	Null/Low-Grade AE N (%) 34 (35.4)	High-Grade AE N (%) 62 (64.6)	OR (95% CI)	*P value*
***ABCC5* rs562**	Codominant	T/T	14 (41.2%)	24 (38.7%)	1.00	0.063
		T/C	19 (55.9%)	27 (43.5%)	0.83 (0.34-2.00)	
		C/C	1 (2.9%)	11 (17.7%)	6.42 (0.75-55.08)	
	Dominant	T/T	14 (41.2%)	24 (38.7%)	1.00	0.810
		T/C-C/C	20 (58.8%)	38 (61.3%)	1.11 (0.47-2.60)	
	Recessive	T/T-T/C	33 (97.1%)	51 (82.3%)	1.00	0.021
		C/C	1 (2.9%)	11 (17.7%)	7.12 (0.88-57.70)	
	Overdominant	T/T-C/C	15 (44.1%)	35 (56.5%)	1.00	0.250
		T/C	19 (55.9%)	27 (43.5%)	0.61 (0.26-1.41)	
	Log-additive	---	---	---	1.49 (0.78-2.84)	0.220
***ABCC5* rs939336**	Codominant	A/A	14 (41.2%)	26 (41.9%)	1.00	0.057
		A/G	19 (55.9%)	25 (40.3%)	0.99 (0.36-2.73)	
		G/G	1 (2.9%)	11 (17.7%)	3.29 (0.83-12.98)	
	Dominant	A/A	14 (41.2%)	26 (41.9%)	1.00	0.940
		A/G-G/G	20 (58.8%)	36 (58.1%)	1.36 (0.51-3.60)	
	Recessive	A/A-A/G	33 (97.1%)	51 (82.3%)	1.00	0.021
		G/G	1 (2.9%)	11 (17.7%)	7.12 (0.88-57.70)	
	Overdominant	A/A-G/G	15 (44.1%)	37 (59.7%)	1.00	0.180
		A/G	19 (55.9%)	25 (40.3%)	0.58 (0.25-1.36)	
	Log-additive	---	---	---	1.70 (0.90-3.20)	0.300
***ABCC5* rs3749445**	Codominant	C/C	14 (41.2%)	25 (40.3%)	1.00	0.058
		C/T	19 (55.9%)	26 (41.9%)	0.77 (0.32-1.85)	
		T/T	1 (2.9%)	11 (17.7%)	6.16 (0.72-52.80)	
	Dominant	C/C	14 (41.2%)	25 (40.3%)	1.00	0.940
		C/T-T/T	20 (58.8%)	37 (59.7%)	1.04 (0.44-2.43)	
	Recessive	C/T-C/T	33 (97.1%)	51 (82.3%)	1.00	0.021
		T/T	1 (2.9%)	11 (17.7%)	7.12 (0.88-57.70)	
	Overdominant	C/C – T/T	15 (44.1%)	36 (58.1%)	1.00	0.190
		C/T	19 (55.9%)	26 (41.9%)	0.57 (0.25-1.33)	
	Log-additive	---	---	---	1.43 (0.75-2.70)	0.270
***ABCC5* rs3792585**	Codominant	A/A	14 (41.2%)	26 (41.9%)	1.00	0.051
		A/G	19 (55.9%)	25 (40.3%)	0.71 (0.29-1.71)	
		G/G	1 (2.9%)	11 (17.7%)	5.92 (0.69-50.70)	
	Dominant	A/A	14 (41.2%)	26 (41.9%)	1.00	0.940
		A/G-G/G	20 (58.8%)	36 (58.1%)	0.97 (0.41-2.27)	
	Recessive	A/A-A/G	33 (97.1%)	51 (82.3%)	1.00	0.021
		G/G	1 (2.9%)	11 (17.7%)	7.12 (0.88-57.70)	
	Overdominant	A/A -G/G	15 (44.1%)	37 (59.7%)	1.00	0.140
		A/G	19 (55.9%)	25 (40.3%)	0.53 (0.23-1.24)	
	Log-additive	---	---	---	1.37 (0.73-2.58)	0.330

AE, Adverse Events; OR adjusted by sex, age at diagnosis, WBC count in peripheral blood, gene rearrangement and chemotherapy protocol. A value of $p \leq 0.05$ was considered statistically significant.
